# Measurement Error Analysis and Thermal Degradation Kinetic Model Improvement for Thermogravimetric Analyzers

**DOI:** 10.3390/polym17172390

**Published:** 2025-09-01

**Authors:** Guixiang Xie, Yaqi Lu, Xiaochun Lu, Zhusen Zhang, Shuidong Lin

**Affiliations:** 1 Fujian Provincial Key Laboratory of Clean Energy Materials, Longyan Nonferrous Metal Industry Research Institute, College of Chemistry and Material Science, Longyan University, Longyan 364012, China; 13600985615@139.com (Y.L.); 82007001@lyun.edu.cn (X.L.); 81991006@lyun.edu.cn (Z.Z.); 2 Fujian Key Laboratory of Photoelectric Functional Materials, Huaqiao University, Xiamen 361021, China

**Keywords:** thermogravimetric analysis, heating rate, SGP membrane, degradation kinetics, service lifetime

## Abstract

Thermogravimetric analysis (TGA) has been extensively applied in polymeric characterization and quality inspection, facilitating in-depth investigations of the microstructural thermal response characteristics of polymers, including thermal stability, composition analysis, and thermal decomposition mechanisms. Here, the impacts of six factors on the TG thermal analysis curves obtained during operation are systematically examined while analyzing their causes and recommending solutions. Furthermore, the thermal degradation kinetics of an ionomer formed by neutralizing an ethylene–methacrylic acid copolymer with metal ions (SGP membrane) used in laminated tempered glass is analyzed using the Arrhenius equation, Ozawa–Flynn–Wall hypothesis and Kissinger method. Kinetic parameters at 5% degradation are fitted and used to predict the service lifetime of the SGP membrane. The results indicate that the SGP membrane sample exhibits activation energy *Ea* = 136.90 kJ/mol, reaction order *n* = 1.65 and pre-factor *A* = e^25.93^. It can be seen that the service lifetime of the SGP membrane sample is 16 years at 80 °C and 1.65 years at 100 °C.

## 1. Introduction

Thermogravimetric analysis (TGA) is a high-precision research tool that characterizes the mass changes of materials with temperature during the heating process under programmed temperature control and a set atmosphere. It has the advantages of good repeatability, high sensitivity and precise control of the thermal process [1,2,3,4]. Moreover, TGA has the advantages of fast analysis speeds, simple operation, low sample consumption and the ability to comprehensively study the thermal decomposition temperatures and material composition ratios of polymer components [5,6,7,8]. The International Confederation for Thermal Analysis and Calorimetry (ICTAC) has proposed a method for kinetic calculations using thermal analysis data [9]. It has been widely applied in the research of polymer components [10,11,12]. However, several factors, such as the heating rate, atmosphere, gas flow rate, crucible capping option and so on, affect the test results of TGA technology [13]. In addition, the dynamic nature of the temperature and the balance characteristics contribute to the complexity of the factors affecting the thermogravimetric curve. Recent research on existing thermogravimetric analysis technology generally only discusses the heating rate, the atmosphere and the influence of samples on the test results [14,15,16,17], and there is seldom comprehensive elaboration on all aspects of the influencing factors in thermogravimetry. In this study, operational issues including six factors (baseline drift, heating rate, different atmospheres, gas flow rates, crucible capping options and sample quality) within the instrument were systematically investigated in order to obtain an accurate and repeatable TG curve.

Ionomers comprise a distinct category of polymers characterized by the presence of a minor fraction (typically 5–15 mol%) of ionic groups along their chains. These ionic groups are typically cross-linked through metal ions to form ionic clusters, which affect the mechanical and thermal properties of the material. The copolymer of ethylene and methacrylic acid belongs to the family of ionomers. Its structure and performance combine the flexibility of polymers with the cross-linking characteristics of ionic bonds, and it is widely used in industry. An SGP membrane is an ionomer formed by neutralizing an ethylene–methacrylic acid copolymer with metal ions, representing an important branch of ionic polymers. Its core advantage lies in balancing heat resistance, mechanical properties and processability through the physical cross-linking of ion clusters, making it particularly suitable for scenarios that require high temperature resistance, high toughness and sealing performance. Therefore, studying the thermogravimetric properties of ionomers is of great significance for the analysis of their service life and application scenarios.

In recent years, researchers have conducted in-depth studies on ionomers through thermogravimetric analysis, analyzing the mixing modes of blends within ionomers, the content of certain components, the stability of polymers, the effects of diffusion on polymerization and so on [18,19,20,21,22]. Copolymers with SGP membranes have been widely used in the design of laminated tempered glass and are accepted by the majority of users because of their high transparency, low fog and excellent weather resistance. However, similarly to most polymer materials, they easily decompose under high-temperature conditions, resulting in reduced mechanical properties, which limit their applications. Therefore, using thermogravimetric analysis to study the thermal stability and degradation kinetics and predict the service life of the film is of great significance for its application and safety, providing support and significant guidance for the systematic exploration of new polymer materials with excellent properties.

## 2. Experimental Section

### 2.1. Materials

NaOH and ethylene–methacrylic acid copolymer (EMAA) were purchased from Aladdin Technology Co., Ltd., Shanghai, China. Nitrogen and compressed air gases were purchased from Riping Industry and Trade Co., Ltd., Anxi, China. All materials were used as received, without purification. Preparation of SGP membrane: EMAA and NaOH solution were proportionally fed into a gravimetric feeder under continuous nitrogen purging. The neutralization reaction was completed in the front section of the extruder (temperature controlled at 80–120 °C), and the neutralized ionomer melt was extruded into a 1-mm-thick film through a die; then, the SGP membrane (an ionomer formed by neutralizing the ethylene–methacrylic acid copolymer with metal ions) was obtained.

### 2.2. Testing

Thermogravimetric analysis data were obtained by a synchronous thermal analyzer (STA) (STA449F3, German Netzsch Instrument Manufacturing Co., Ltd., Selb, Germany) heated from 20 to 1200 °C at a heating rate of 3, 5, 10, 20 K·min^−1^ under an air and nitrogen compressed gas atmosphere, in a crucible with or without a cover.

**Figure 1:**
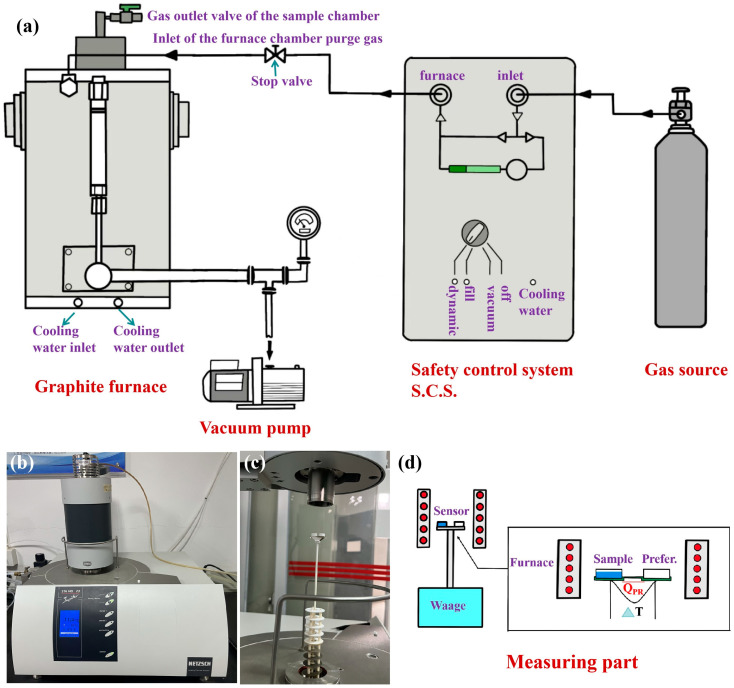
Schematic diagram of the STA449F3 instrument. (**a**) Gas connection system; (**b**) test unit; and (**c**,**d**) upper-mounted balance measurement structure.

Figure 1 shows the structural diagram of the thermogravimetric analyzer (STA 449F3). The gas connection system consists of three parts—the graphite furnace, the safety control system and the gas source—as represented in Figure 1a. Figure 1b,c shows photos of the instrument mainframe and the alumina bracket. The mechanism diagram of the top-mounted balance weighing system is shown in Figure 1d. As can be seen from the figure, the gas enters the heating furnace after flow control and cooling circulation to maintain the stability inside the furnace. The TG and DSC values are obtained by comparing the mass and heat differences between the reference crucible and the sample crucible through the bracket.

### 2.3. Mathematical Modeling of Thermogravimetric Degradation Kinetics

In the thermogravimetric test, the degradation degree of the sample at a certain moment can be expressed by the degradation rate *α*, which refers to the ratio of the mass lost at a certain moment to the final mass lost [23]. The degradation rate *α* of the sample at a certain moment can be expressed as follows [24]:

(1) $$\alpha =\frac{m_{i}-m}{m_{i}-m_{f}}$$

*m_i_*, *m* and *m_f_* represent the initial mass, the mass at a certain time and the final mass of the sample, respectively. By integrating the degradation rate over time (*dα/dt*), the relationship between the reaction rate coefficient *k* and *α* can be obtained [25]:

(2) $$\frac{d\alpha }{dt}=\kappa \cdot f(\alpha )$$

It is known that the *k* in the Arrhenius equation [26,27] is

(3) $$\kappa =A\cdot \exp(\frac{-E_{a}}{RT})$$

*A* refers to the pre-factor (min^−1^), *Ea* is the activation energy of the degradation reaction (kJ·mol^−1^), *R* is the gas constant (R = 8.314) and *T* is the absolute temperature (K). Substituting this into Equation (4), the following relation can be obtained [28]:

(4) $$\frac{d\alpha }{dt}=A\cdot \exp(-\frac{E_{a}}{RT}){\left(1-\alpha \right)}^{n}$$

The sample is heated at *a* constant speed under program control, and the heating rate can be expressed as *β* ($\beta =\frac{dT}{dt}$), derived in Formula (5) as follows:

(5) $$\frac{d\alpha }{{\left(1-\alpha \right)}^{n}}=\frac{A}{\beta }\cdot \exp(-\frac{E_{a}}{RT})dT$$

Formula (5) is the basic equation for the degradation kinetics of samples and one of the most commonly used expressions. The two sides of the equation can be integrated as follows:

(6) $$g(\alpha )=\int \frac{1}{{\left(1-\alpha \right)}^{n}}d\alpha =\frac{A}{\beta }\int \exp(-\frac{E_{a}}{RT})dT=\frac{AE_{\alpha }}{\beta R}p(x)$$

where $x=\frac{E_{a}}{RT}$. According to the Ozawa–Flynn–Wall method [29,30], lnp(x)=−1.052x−5.330, 20 < *x* < 60; then, Formula (6) can be rewritten as

(7) $$\ln g(\alpha )=\ln\frac{AE_{\alpha }}{\beta R}-1.052\frac{E_{\alpha }}{RT}-5.330$$

Here, A and R are constant, and g (*α*) is also constant for the degradation rate *α* at a certain time. Then, Formula (7) can be changed to [31]

(8) $$\ln\beta =-1.052\frac{E_{\alpha }}{RT}+\ln\frac{AE_{\alpha }}{Rg(a)}-5.330$$

## 3. Results

### 3.1. Influencing Factors for Thermogravimetric Analysis

Baseline drift refers to the phenomenon where, during the heating process of the instrument, the mass of the sample does not increase or decrease, but the recorded curve shows a change in mass. The baseline drift in TGA is primarily influenced by buoyancy effects, gas convection and the thermal expansion of the support material [32]. Buoyancy effects occur when an increase in the furnace temperature leads to a decrease in the gas density, resulting in reduced air buoyancy and an increased sample mass. Gas convection by heating describes how rising temperatures within the furnace generate airflow that exerts a force on the support material, thereby affecting the sample quality. Furthermore, the thermal expansion of the support material can also impact the sample quality. To mitigate the influence of baseline drift on the experimental results and to identify suitable baselines for correction, it is common practice to test for baseline drift prior to conducting experiments (as depicted in Figure S1). Deducting baseline drift ensures greater accuracy of the data.

**Figure 2:**
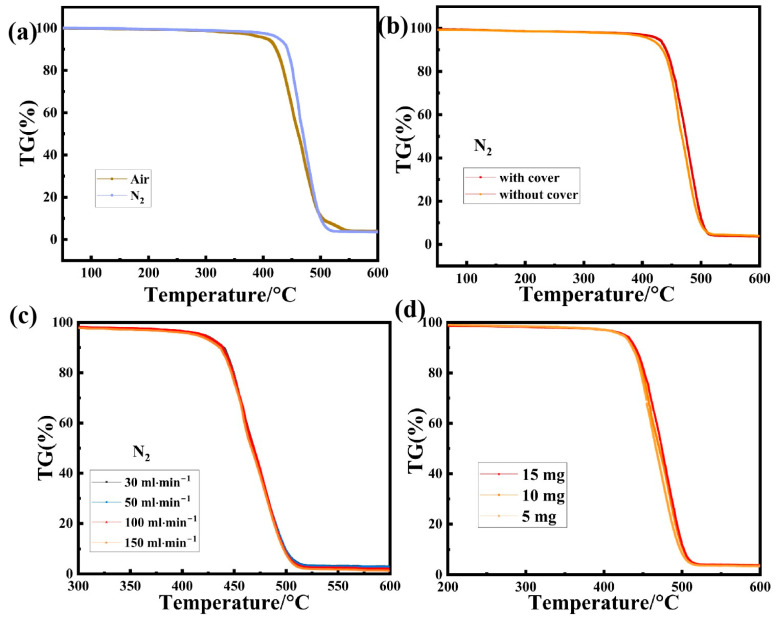
Influencing factors in thermogravimetric analysis of SGP membrane: (**a**) under different atmospheric purging; (**b**) with crucible cover before and after calcination; (**c**) flow rates of purge gas at baseline; and (**d**) sample mass in TG test.

The effects of various atmospheres on the TG baseline and thermal decomposition of SGP membranes are illustrated. As can be seen from Figure S2, the baseline drift is smaller in the air atmosphere than in the nitrogen atmosphere. As seen in Figure 2a, when the SGP membrane undergoes thermal degradation with increasing temperatures in the air and nitrogen atmospheres, the initial degradation temperatures are 416.8 °C and 444.4 °C, while the final degradation temperatures are 500.8 °C and 499.6 °C, respectively. This indicates that the SGP membrane experiences earlier thermal decomposition in the air atmosphere than under nitrogen, so the selection of the atmosphere can help in the separation of the weightless step. The various atmospheres introduced in the system are as follows: a nitrogen (N_2_) atmosphere, which is an inert atmosphere suitable for most materials (although some metal materials may react at high temperatures); an argon (Ar) atmosphere, which is another inert atmosphere, primarily used for the high-temperature testing of metal materials; a helium (He) atmosphere, which is also an inert atmosphere, occasionally utilized in STA low-temperature furnace and ultra-high-temperature furnace tests due to its excellent thermal conductivity (requires separate sensitivity correction); an air atmosphere, which is a commonly employed oxidizing atmosphere, often used as a purging medium for ceramic oxide samples and organic samples; an oxygen (O_2_) atmosphere, which is a strong oxidizing atmosphere typically employed as a reaction medium. To maintain uniform furnace temperatures throughout the experimental system and to promptly remove the generated gas products, an inert atmosphere is generally selected. However, if it is necessary to study the sample’s behavior under specific atmospheres, a corresponding experimental environment—either an inert or reactive atmosphere—should be selected. For the investigation of the thermal behavior of a sample in a natural atmospheric setting, it is not required to introduce atmospheric gases into the sample chamber; thus, the flow rate can be set to zero or the gas switch can be turned off.

The crucible and its cover influence the TG baseline and thermal decomposition of the SGP membrane, both before and after calcination, as also illustrated. The baseline drift of the crucible after calcination is the smallest, as shown in Figure S3, followed by the crucible without calcination and sealed, and the test baseline drift with the crucible without calcining and sealed is the largest. The thermal decomposition of the SGP membrane (Figure 2b) was also tested with both open and sealed crucibles after calcination, and the initial degradation temperatures were 437.0 °C and 441.7 °C, while the final degradation temperatures were 495.6 °C and 506.4 °C, respectively. The residual masses were 3.92% and 3.98%, respectively. The results show that the thermal decomposition temperature of the opened sample was lower, and the residual mass was smaller, indicating a more complete reaction. In general, there are two main types of crucibles used in TG experiments: open crucibles and sealed crucibles. The advantages of using a crucible cover include an improvement in the temperature distribution within the crucible, which promotes uniformity in the temperature distribution in the reaction system. In addition, it effectively reduces radiation effects and minimizes sample coloration. Furthermore, it prevents light or fine sample powders from becoming airborne or being carried away during vacuum extraction processes. However, it carries certain disadvantages. For instance, it reduces the contact between the reaction atmosphere and the sample, hindering gas–solid reactions (oxidation, reduction, adsorption) to some extent. Moreover, the slow gas removal of the product can lead to higher partial pressure around reactants, potentially affecting the chemical equilibrium between the reaction rate and gaseous-phase products. Furthermore, competitive reaction mechanisms may impact the product composition by altering the weight loss rate of the TG step. For unknown samples, capping is usually selected for safety reasons.

Figure 2c illustrates the impact of the purge gas at varying flow rates (30, 50, 100, 150 mL/min) on the thermal decomposition of SGP membranes. The initial degradation temperatures are 444.4 °C, 441.8 °C, 441.9 °C and 441.5 °C, while the final degradation temperatures are 500.7 °C, 504.4 °C, 499.9 °C and 499.4 °C, respectively. The residual masses are 3.1%, 2.8%, 2.3% and 1.3%, respectively. The results indicate that, as the gas flow rate increases, the baseline drift decreases (Figure S4), and the thermogravimetric temperature of the SGP membrane also decreases. This suggests that a higher flow rate facilitates the faster removal of gaseous products, thereby promoting the progression of the reaction and potentially lowering the temperature of thermal decomposition. However, it is important to avoid excessively high flow rates in the experimental atmosphere under which TGA is carried out, as it can result in incomplete decomposition and eventually affect the shape of the thermal analysis curve by carrying away lighter samples without sufficient time for complete decomposition. Conversely, an overly low flow rate may hinder the timely discharge of decomposition products, often leading to increased decomposition temperatures and potential impacts on the reaction mechanisms in severe cases. Under different “inert atmospheres”, the reaction temperatures may differ and even alter the reaction mechanism, thereby affecting the weight loss proportions. When decomposed product gases are used as purge atmospheres, the chemical equilibrium is affected, which results in an increase in the reaction temperature. Therefore, the selection of an appropriate atmosphere and the regulation of the gas flow can sometimes serve as techniques for step separation. For samples that release pollution-causing products, it is recommended to use a larger purge airflow, such as 100 mL·min^−1^ or greater. 

The sample quality primarily affects the heat conduction (temperature gradient) and diffusion of volatile products (escaping gas), thereby influencing the shape of the TG curve. For instance, a smaller sample size reduces the temperature gradient, resulting in lower and more accurate measurements of characteristic temperatures. It also facilitates the diffusion of gas products and minimizes reverse reactions at chemical equilibrium. In contrast, a larger sample size enhances the differential scanning calorimetry sensitivity but widens the peak shape, shifts the peak temperature to higher values, causes adjacent peaks (platforms) to merge together and decreases the peak separation ability. Moreover, larger samples also exhibit a greater temperature gradient and slightly poorer gas product diffusion.

Figure 2d illustrates the impact of variations in the mass of the SGP membrane on TG testing. When the masses of the SGP membranes are 5, 10 and 15 mg, respectively, the initial degradation temperatures of thermogravimetric decomposition are 439.8 °C, 440.2 °C and 444.3 °C, respectively, and the residual masses are 3.2%, 3.8% and 3.6%, respectively. A greater sample mass leads to the displacement of the weight loss steps due to increased temperature gradient differences within the sample. Therefore, ensuring an appropriate sample dosage is crucial in obtaining optimal curves. Importantly, it is crucial to maintain the amount of sample within the sensitivity range of the TG analyzer. In cases where samples are severely decomposed, it is recommended to minimize their quantities during TG experiments.

**Figure 3:**
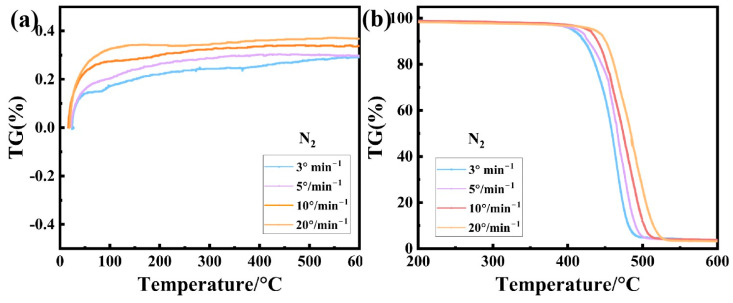
Effects of different heating rates on (**a**) TG baseline and (**b**) thermogravimetric process of SGP membrane.

The influence of various heating rates (3, 5, 10, 20 K·min^−1^) on the TG baseline (Figure 3a) and the thermal decomposition of the SGP membrane sample under a nitrogen atmosphere (Figure 3b) is illustrated. When the heating rates are 3, 5, 10 and 20 K·min^−1^, respectively, the initial decomposition temperatures are 437.2 °C, 442.8 °C, 445.0 °C and 481.9 °C, respectively; the final decomposition temperatures are 480.2 °C, 490.0 °C, 503.4 °C and 509.1 °C; and the residual masses are 3.9%, 3.5%, 3.6% and 3.3%, respectively. The figure illustrates that different heating rates exert distinct impacts on the TG curves across the same nitrogen atmospheres. A higher heating rate leads to greater baseline drift and more significant thermal hysteresis during the thermal decomposition of the SGP membrane, resulting in higher initial and final degradation temperatures in the TG curves. With the increase in the heating rate of the SGP membrane, the weight loss peak is higher, because there is a delay effect in the decomposition process. The molecular chain movement is intensified during the heating process, but the thermal conductivity of the membrane is small, so the energy cannot be transmitted in a timely manner when the heating is fast, causing a certain lag, and the final outcome is that the weight loss temperature increases with the heating rate. In addition, when the temperature rises to 400 °C, the thermal degradation rate of the SGP membrane will increase with the increase in the heating rate. After 400 °C, with the acceleration of the heating rate, the thermal degradation trend becomes slower, and the degradation rate in the 20 K/min temperature curve is significantly slower than that in the 3 K/min temperature curve. This is because the sample can be fully decomposed at a slow heating rate, while the decomposition time is short at the same temperature at a higher rate, resulting in inadequate decomposition.

Therefore, it is extremely important to select an appropriate heating rate for sample testing. A higher heating rate can effectively enhance the instrument’s sensitivity; nonetheless, excessively high rates may result in more pronounced thermal hysteresis, leading to elevated initial and final temperatures in the TG curve and consequently introducing errors into the measurement results. In practical applications, it is crucial to comprehensively consider both the transformation nature and instrument sensitivity when selecting an appropriate temperature scanning rate. The most commonly employed temperature scan rate for TG experiments is typically 10 °C·min^−1^.

### 3.2. Prediction of Service Lifetime of SGP Membrane

**Figure 4:**
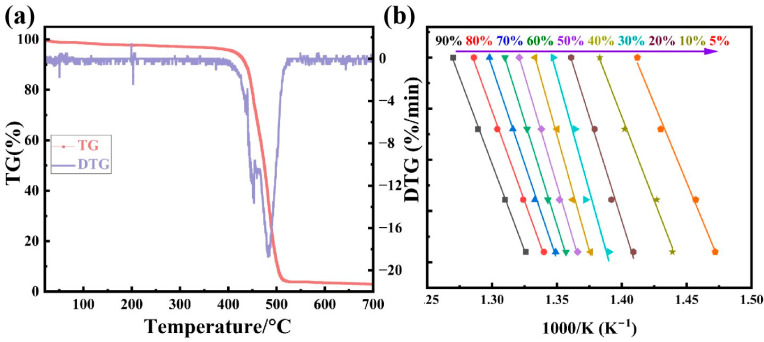
(**a**) TG and DTG curves of SGP membrane with temperature scan rate of 10 °C·min^−1^; (**b**) curve of lnβ change with 1000/T.

The TG and DTG curves of the SGP membrane with a heating rate of 10 K·min^−1^ are shown in Figure 4a. The initial thermal decomposition temperature (T5%) of the SGP membrane is 426.5 °C, and it is completely decomposed at about 700 °C, with a residual rate of about 2.94%, indicating that the material exhibits good thermal stability. In addition, it can be found from the DTG curve that the thermal degradation behavior of the SGP membrane is mainly divided into three stages: first, the volatilization of the ionic bonds between metal ions and carboxylic acid groups occurs, and some weak chain segments (such as side chains or ends) begin to break, generating small-molecule alkenes when the temperature is between 387.6 °C and 439.8 °C. Then, the random breakage of macromolecular chains occurs, and the C-C bonds of ethylene segments break on a large scale, generating a large number of alkyl radicals when the temperature is between 439.8 °C and 455.2 °C. The remaining small-molecule chains further break into small-molecular gases (such as H_2_, CO, CH_4_), and, at the same time, the carboxyl groups are completely removed at 455.2–522.0 °C, ultimately leaving a small amount of carbon residues [33,34]. The temperatures (°C) corresponding to different degradation rates of the SGP membrane under different heating rates can be obtained from Figure 3b. According to the temperatures, the linear relationship between lnr and 1/T at different heating rates can be obtained (Figure 4b). The linear fitting degree R^2^ of each straight line was found to be greater than 99.7%, indicating that the thermogravimetric data conformed to the assumptions of the kinetic equation on which they were based. We next observe the lnr and 1/T values corresponding to the starting and ending points of the curves with different heating rates. When the heating rate is small, the temperature corresponding to the starting point is relatively low, the weight loss rate is small, and the starting point gradually increases with the increase in the heating rate, indicating that the heating rate has an impact on the initial stage of the reaction. The slope differences in curves with different heating rates reflect the variation in the activation energy with the heating rate. The slope difference between the low-temperature and high-temperature sections is relatively small, indicating that the activation energy is relatively stable and the reaction mechanism is not greatly affected by the heating rate. The slopes in the moderate-temperature section vary significantly, indicating that the reaction path may have changed at different heating rates.

**Figure 5:**
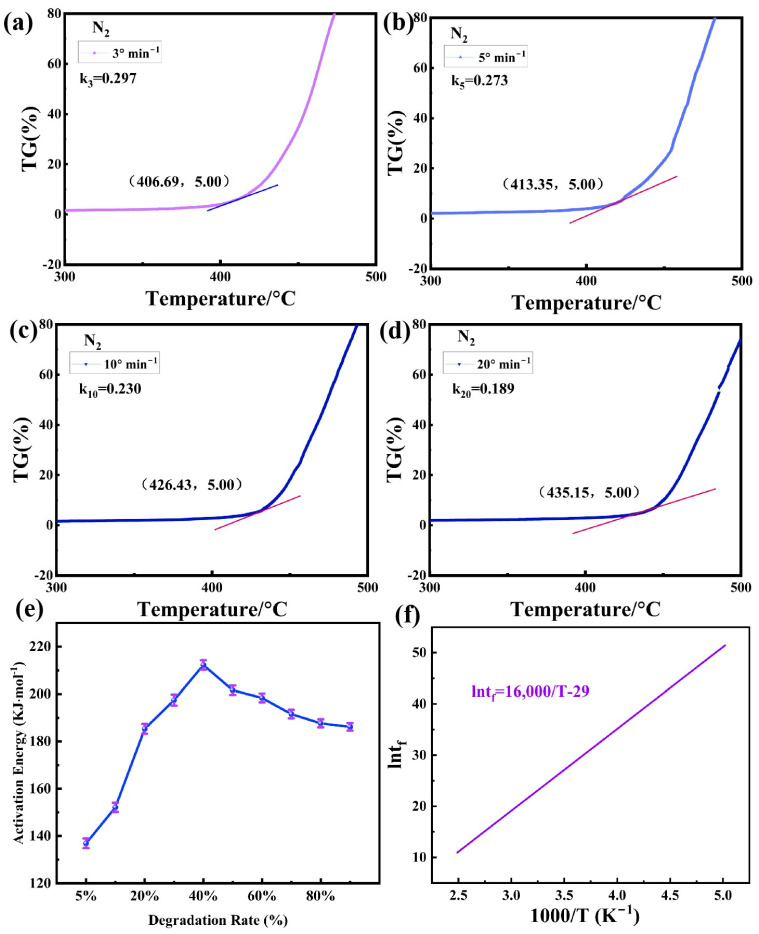
(**a**–**d**) dα/dt values of each heating rate when the degradation rate is 5%; (**e**) degradation activation energy at different degradation rates of the SGP membrane; (**f**) variation relationship among the service lifetime of the SGP membrane and the temperature.

According to Formula (8), the activation energy (Ea) at any degradation temperature (Figure 5e) and different degradation rates can be calculated via the linear relationship between lnβ and 1/T at different heating rates, as shown in Figure 4b [35]. It can be seen from Figure 5e that the degradation and Ea of the SGP membrane increases with the degradation degree. The ionic bond breaking between metal ions and carboxylic acid groups requires relatively high energy, resulting in an increase in activation energy. After the cross-linking is broken, the resistance to the decomposition of the main chain decreases, resulting in a decline in the activation energy. When most of the main chain breaks, the remaining fragments may contain high-concentration carboxylic acid groups or polar fragments, and the intermolecular interaction is enhanced again, leading to the stabilization of the activation energy. This is consistent with the thermogravimetric behavior discussed earlier in the air atmosphere.

For polymer materials, the service lifetime of the material *t_f_* (min) is often determined by predicting the failure time at a particular level of performance, where the temperature at 5% heat loss in TG experiments as the failure standard of the polymer material has been widely used to evaluate the service life of the material [36]. Integrating the two sides of Formula (4), we have

(9) $$t_{f}=\frac{1-{\left(1-\alpha \right)}^{1-n}}{A(1-n)}\exp\frac{E_{a}}{RT}\;(n\neq 1)$$

(10) $$\mathrm{or}\;t_{f}=\frac{-\ln\left(1-\alpha \right)}{A}\exp\frac{E_{a}}{RT}\;(n=1)$$

The reaction series *n* in Equation (9) can be found via the Kissinger method [27] using the following formula:

(11) $$S=\frac{{\left[d^{2}\alpha /dt^{2}\right]}_{L}}{{\left[d^{2}\alpha /dt^{2}\right]}_{R}}$$

(12) n=1.88×S

where the subscripts *L* and *R* in Equation (11) represent the left and right peaks of the second-order differential curve of TG (DDTG), respectively, and *d*^2^*α*/*dt*^2^ can be obtained through the DDTG curve. Thus, the reaction series *n* and its average value for the SGP membrane can be calculated. According to the activation energy *Ea* and different heating rate values of dα/dt (Figure 5a–d) at 5% degradation, the value of ln*A* is obtained from the model equation of the degradation dynamics, i.e., Formula (5), as shown in Table 1.

As can be seen from Table 1, the average *n* in the thermal degradation of this SGP membrane system is 1.65, which indicates a multistage degradation reaction, and the degradation process is relatively complex. The degradation activation energy reaches 136.90 kJ/mol (calculated using a mathematical method), which indicates a strong reaction barrier and shows good heat resistance stability. The relationship between the service lifetime t_f_ and temperature can be obtained via the kinetic parameters in Table 1 and Equation (9) [37,38]:

(13) $${\mathrm{lnt}}_{f}=16000/T-29$$

Drawing this relation from Equation (13) gives a straight line, as shown in Figure 5f. 

As can be seen from Formula (13) and Figure 5f, the service lifetime of the SGP membrane is highly dependent on the temperature, and its service lifetime decreases sharply with the increase in the environmental temperature. After calculation, the service lifetime of the SGP membrane at 80 °C is over 16 years, while the service lifetime at 100 °C is reduced to 1.8 years.

## 4. Conclusions

This study discusses the principles and influential factors of TGA testing, including baseline drift, the heating rate, the atmosphere, the gas flow rate, the presence or absence of a crucible cap and the sample quality. Finally, TGA is employed to test the thermal stability of an SGP membrane, and the results show that the thermal degradation behavior of the SGP membrane is mainly divided into three stages, indicating that the material has good thermal stability. The basic equations for the degradation kinetics, degradation activation energy *Ea* and service lifetime *t_f_* of the SGP membrane are obtained via the Arrhenius equation, Ozawa–Flynn–Wall hypothesis and Kissinger method. The *Ea* of the SGP membrane, reaction series *n* and forward factor *A* at a 5% degradation rate are *Ea* = 136.9 kJ/mol, *n* = 1.65 and *A* = e^25.93^, and the relationship between the service life and temperature is ln*t_f_* = 16,000/*T* − 29. It can be seen that the service lifetime of the SGP membrane is over 16 years at 80 °C and reduces to 1.6 years at 100 °C.

## Figures and Tables

**Table 1:** Degradation kinetic parameters at 5% weight loss of SGP membrane [37,38].

Heating Rate β (k·min^−1^)	*n*	lnA
3	1.64	25.60
5	1.68	25.58
10	1.63	25.85
20	1.63	26.68
Mean value	1.65	25.93
Standard deviation	0.02	0.51

## Data Availability

Data will be made available on request.

## Supplementary Materials

The following supporting information can be downloaded at: https://www.mdpi.com/article/10.3390/polym17172390/s1, Figure S1: Baseline testing of STA; Figure S2: Baseline under different atmospheric purging; Figure S3: Effects of crucible and its cover before and after calcination on baseline; Figure S4: Effects of different flow rates of purge gas on baseline.
