# Supplementary material for: Measurement Error Analysis and Thermal Degradation Kinetic Model Improvement for Thermogravimetric Analyzers
Source: Polymers (Basel). 2025 Sep 1;17(17):2390. doi: 10.3390/polym17172390 (PMC12431055; doi:10.3390/polym17172390)
Supplement: Supplementary file 1 [file polymers-17-02390-s001.zip › polymers-3682019-supplementary.pdf]

Thermogravimetric Analysis: Contructing a Kinetic Model to Decode the Lifecycle Mechanisms of X-SGP Membrane

Guixiang Xie [1 2*], Yaqi Lu [1], Xiaochun Lu [1], Zhusen Zhang [1] and Shuidong Lin [1*]

[1] Fujian Provincial Key Laboratory of Clean Energy Materials (Longyan University); Longyan Nonferrous Metal Industry Research Institute (Longyan University); College of Chemistry and Material Science, Longyan University, Longyan 364012, P. R. China;

[2] Fujian Key Laboratory of Photoelectric Functional Materials, Huaqiao University, Xiamen, Fujian 361021, P. R. China;

*Corresponding authors. E-mail addresses: 82010012@lyun.edu.cn (G. Xie), 81991006@lyun.edu.cn (Z. Zhang).

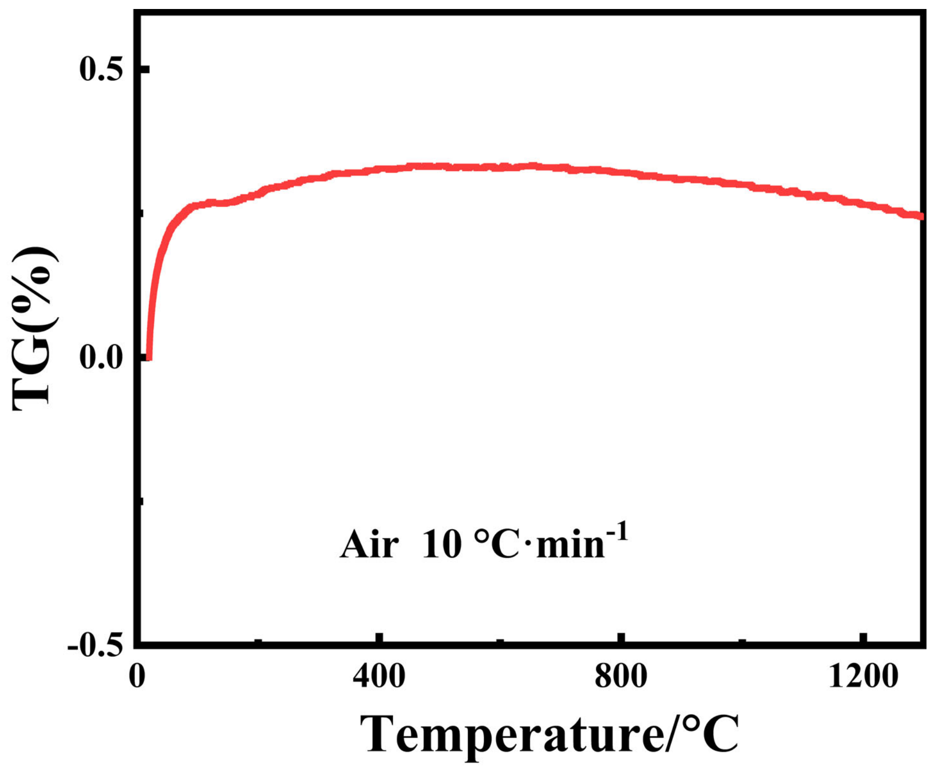

Figure S1. Baseline testing of STA

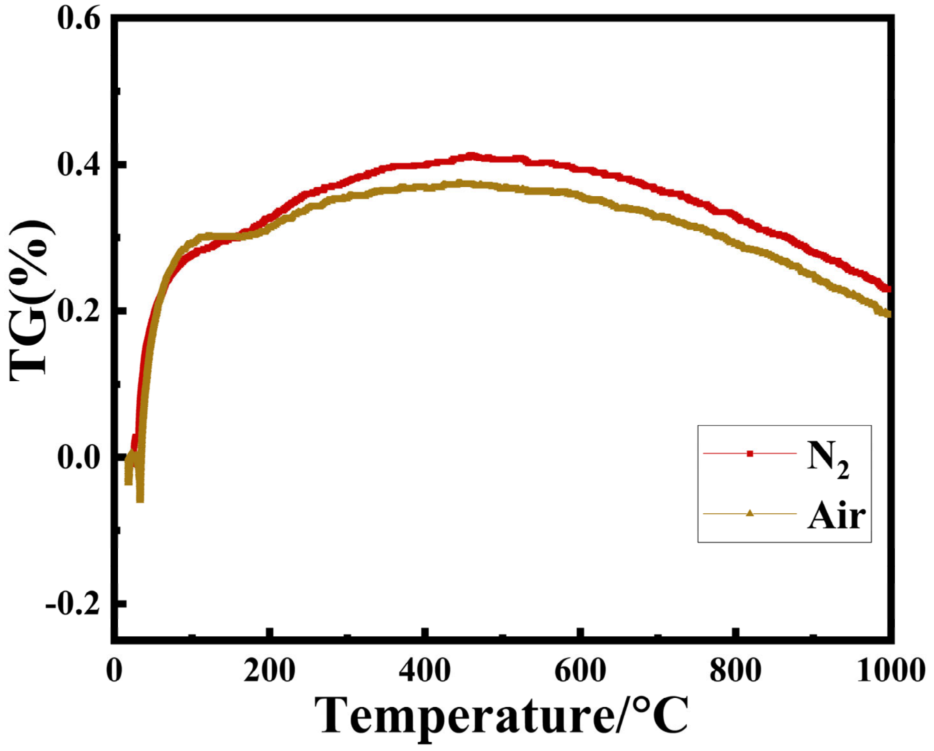

Figure S2. Baseline under different atmosphere purge

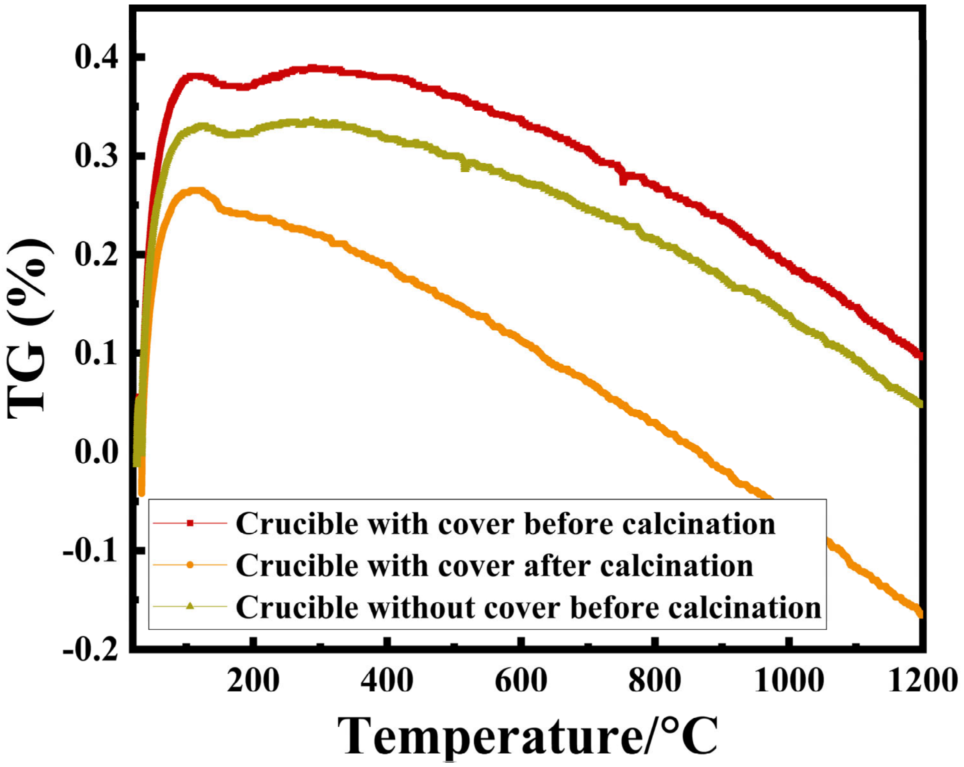

Figure S3. Effect of crucible and its cover before and after calcination on baseline

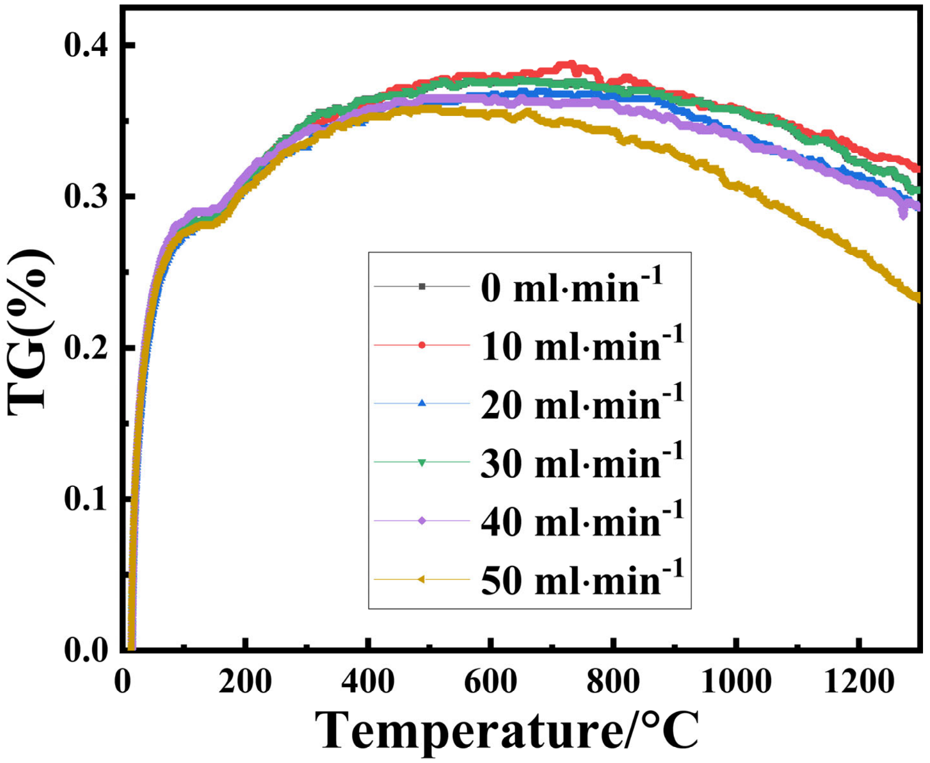

Figure S4. Effects of different flow rates of purge gas on baseline
